# Estimation of coalescence probabilities and population divergence times from SNP data

**DOI:** 10.1038/s41437-021-00435-8

**Published:** 2021-05-01

**Authors:** Kristy Mualim, Christoph Theunert, Montgomery Slatkin

**Affiliations:** 1 Department of Genetics, Stanford University School of Medicine, Stanford, CA USA; 2 Department of Integrative Biology, University of California, Berkeley, CA USA; 3 Present Address: mewedo Ltd., Leipzig, Germany

**Keywords:** Rare variants, Evolutionary genetics

## Abstract

We present a method called the *G*(*A*|*B*) method for estimating coalescence probabilities within population lineages from genome sequences when one individual is sampled from each population. Population divergence times can be estimated from these coalescence probabilities if additional assumptions about the history of population sizes are made. Our method is based on a method presented by Rasmussen et al. (2014) to test whether an archaic genome is from a population directly ancestral to a present-day population. The *G*(*A*|*B*) method does not require distinguishing ancestral from derived alleles or assumptions about demographic history before population divergence. We discuss the relationship of our method to two similar methods, one introduced by Green et al. (2010) and called the *F*(*A*|*B*) method and the other introduced by Schlebusch et al. (2017) and called the TT method. When our method is applied to individuals from three or more populations, it provides a test of whether the population history is treelike because coalescence probabilities are additive on a tree. We illustrate the use of our method by applying it to three high-coverage archaic genomes, two Neanderthals (Vindija and Altai) and a Denisovan.

One of the goals of population genetics is to estimate the divergence time of isolated populations. We will review several methods that have been proposed and present a new method that is closely related to two existing methods. We will emphasize the assumptions made when using different methods. It will be useful to make the distinction between estimating coalescence probabilities within populations and estimating population divergence times. We will also introduce a test for a treelike population history based on our method.

For distantly related populations, the numbers of mutational differences between sequences indicate relative times of divergence. Relative times are converted to absolute times by assuming a mutation rate. This method traces to Zuckerkandl and Pauling (1962, 1965) and has been used and refined extensively. This class of methods estimates genomic divergence times. Using it to estimate population or species divergence times assumes that those times are so large that the difference between them can be ignored.

For recently diverged populations, the numbers of mutational differences probably do not provide a reliable estimate of population divergence times both because there may be too few mutations that differentiate populations and because the difference between the genomic and population divergence times may be substantial. To overcome this problem, Green et al. (2010) (in Supplement 14) introduced a method that accounts for the difference between genomic and population divergence. This method was used in later papers from the same group (Meyer et al. 2012; Prüfer et al. 2014, 2017).

The Green et al. (2010) method is applicable when one genome is sampled from each of two populations. It depends on the statistic *F*(*A*|*B*), which is the fraction of sites in population *A* that carry the derived allele when that site is heterozygous in population *B*. Green et al. (2010) showed by simulation that the expectation of *F*(*A*|*B*) decreases roughly exponentially with the separation time of *A* and *B*. The rate of decrease depends on the history of population sizes both in *B* and in the population ancestral to *A* and *B*. Green et al. (2010) estimated population divergence times by interpolating their simulation results.

More recently, Schlebusch et al. (2017), in Section 9.1 of their supplementary materials, introduced a similar method, called the TT method. Their method is based on analytic expressions for the configuration probabilities of SNPs that are polymorphic in the two populations. The TT method assumes that ancestral and derived alleles can be distinguished and the population before divergence was of constant size. The TT method is developed and elaborated on by Sjödin et al. (2020).

In the present paper, we present a new method that is closely related to the *F*(*A*|*B*) and TT methods. We call it the *G*(*A*|*B*) method to emphasize its similarity to *F*(*A*|*B*). Our method is based on a method presented by Rasmussen et al. (2014) to test whether an ancient DNA sequence is from a population directly ancestral to a present-day population. We will show that our method provides a way to test whether the history of three or more populations is accurately represented by a population tree even if the demographic histories of those populations are not known.

## Analytic theory of *F*(*A*|*B*)

**Fig. 1:**
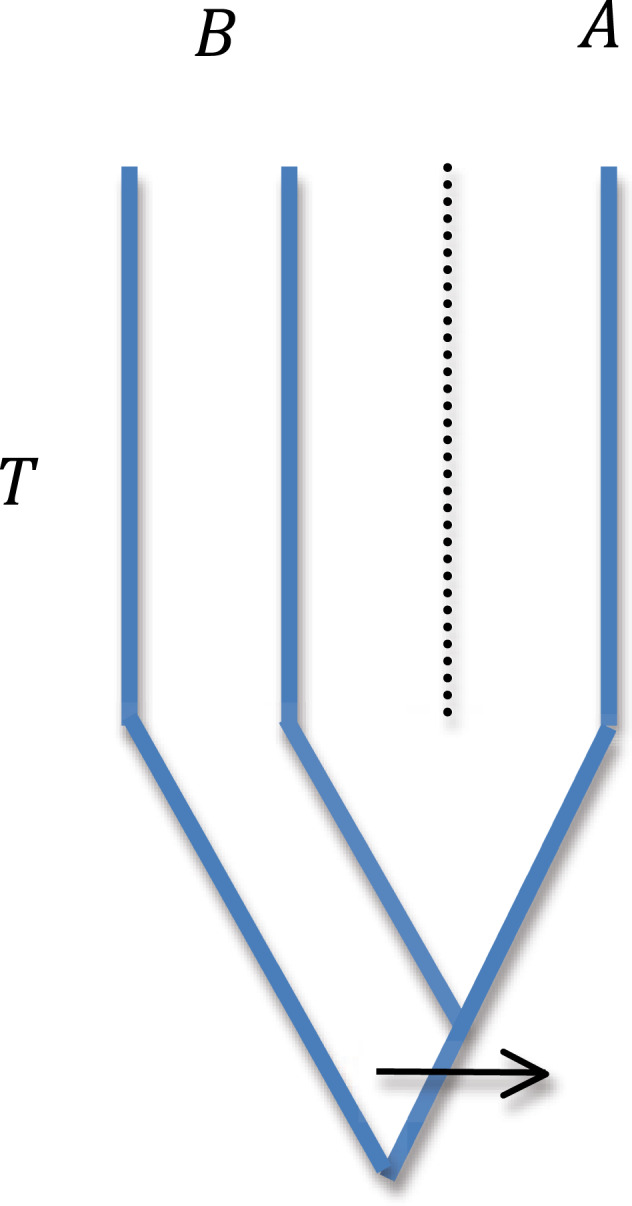
Illustration of the notation used in this paper. Populations *A* and *B* are assumed to have diverged from a common ancestor *T* generations in the past. Two chromosomes from *B* and one from *A* are sampled. A mutation from the ancestral to derived allele at a SNP is assumed to have occurred on the gene tree as shown by the arrow.

Two populations *A* and *B* diverged at time *T* in the past and remained isolated since. Two chromosomes are sampled from population *B* and one from *A*. Let *N*(*t*) denote the population size *t* generations before the present (*t* = 0). Between 0 and *T*, *N*(*t*) is the effective size of population *B*. Before *T*, it is the effective size of the ancestral population. Because only one chromosome is sampled from *A*, the effective size of *A* between 0 and *T* does not matter. If there is no recurrent mutation, *A* carries the derived allele only if one of the two B lineages coalesced with the *A* lineage and there was a mutation on the internal branch of the gene tree, as illustrated in Fig. 1. We calculate the probability of those two events using standard coalescent theory.

The probability of the gene tree shown in Fig. 1 is 2(1 – *c*)/3 where *c* is the probability that the two *B* lineages coalesce between 0 and *T*. The 2/3 reflects the fact that in the ancestral population each pair of lineages is equally likely to coalesce first. The probability that there is coalescence between 0 and *T* is

1 $$c = 1 - \mathop {\prod}\limits_{t = 0}^{T - 1} {\left( {1 - \frac{1}{{2N\left( t \right)}}} \right)} \approx 1 - {\rm{exp}}\left( {{\int}_0^T {\frac{{dt}}{{2N\left( t \right)}}} } \right)$$

where the approximation is accurate when *N*(*t*) is large. If *N* is constant, *c* ≈ 1 – *e*^−*T*/(2*N*)^.

We denote the expected length of the internal branch of the gene tree shown in Fig. 1 by *u*. In general *u* depends on *N*(*t*) in a complicated way but if *N* is constant, *u* = 2*N* (Wakeley 2009). The probability that a mutation occurs on the internal branch is *µu* where *µ* is the per-site mutation rate.

The probability that the two *B* lineages carry different alleles is $2\mu \bar t$, where

2 $$\bar t = {\int}_0^\infty {\frac{1}{{2N\left( t \right)}}} {\rm{exp}}\left[ { - {\int}_0^t {\frac{{dt^{\prime} }}{{2N\left( {t^{\prime} } \right)}}} } \right]$$

is the average coalescence time. Note that $\bar t$ does not depend on *T*. When *N* is constant $\bar t = 2N$.

We denote the probability that *A* carries the derived allele given that the two *B* lineages carry different alleles by *P*(*A*|*B*). We distinguish this probability from the statistic *F*(*A*|*B*) computed from the data. From the rules of conditional probability we obtain

3 $$P\left( {A|B} \right) = \frac{{2\left( {1 - c} \right)}}{3}\frac{{\mu u}}{{2\mu \bar t}} = \frac{{\left( {1 - c} \right)u}}{{3\bar t}}$$

which reduces to *P*(*A*|*B*) = *e*^−*T*/(2*N*)^/3 when *N* is constant. If *N* varies with time, an analytic expression for *P*(*A*|*B*) can be obtained for some functional forms of *N*(*t*), but in practice it may be easier to determine the dependence on *T* by simulation, as was done by Green et al. (2010) and in later papers.

Green et al. (2010) estimated the decrease in *P*(*A*|*B*) with time for several demographic models and then estimated *T* by finding the intersection point with the observed value of *F*(*A*|*B*) with each simulated curve. In Eq. (3), *P*(*A*|*B*) depends on *N*(*t*) both before and after *T* because *u* does.

## Schlebusch et al. (2017) TT method

Another method for estimating population divergence times was presented by Schlebusch et al. (2017) in part 9 of their supplemental materials (pp. 21–23). They call this method the TT method and note that it is related to the concordance methods previously used by Schlebusch et al. (2012) and Skoglund et al. (2011). Schlebusch et al. (2017) assume that two chromosomes are sampled from each population and distinguish nine configurations of the data at each site: O0 (0/0), O1 (1/0), O2 (0/1), O3 (2/0), O4 (0/2), O5 (1/1) O6 (2/1), O7 (1/2), and O8 (2/2), where the numbers before and after the slash are the numbers of derived alleles in the first and second populations respectively. Schlebusch et al. derived the probabilities of each configuration under the infinite sites model with constant mutation rate, arbitrary population size changes after population separation, and constant population size in the ancestral population. These probabilities depend on several parameters: the probabilities of coalescence in the two daughter populations, here called *c*_1_ and *c*_2_ to be consistent with the notation in the previous section, *T*_1_ and *T*_2_ (the population split times for each population scaled by the effective population sizes), *V*_1_ and *V*_2_ (the expected times to coalescence in the two populations, given that they coalesce before the populations split), and *θ*, (the effective size of the ancestral population scaled by the mutation rate). They assume that the numbers of sites in each configuration take their expected values, and they derived expressions for each of the parameters. In particular, they showed that the two coalescence probabilities are given by

4 $$c_1 = \frac{{2m_5}}{{2m_5 + m_6}}$$

5 $$c_2 = \frac{{2m_5}}{{2m_5 + m_7}}$$

where *m*_*i*_ is the observed numbers of sites in configuration O*i*.

Recently, Sjödin et al. (2020) presented a more complete derivation of the TT method and introduced a modification of that method that is similar to the *G*(*A*|*B*) method described below. The new version of the TT method, called the TTo method, assumes that there was an outgroup that diverged from the ancestor of the two populations whose divergence time is being estimated. By restricting the analysis to sites that are polymorphic in the outgroup, the mutation rate is no longer needed. For those sites, Sjödin et al. (2020) derive expressions for the probabilities of coalescence in each of the two populations after they diverge. They also present a test of the hypothesis that the three populations have a history represented by a bifurcating tree. That test is somewhat different from the test of treeness that we present below.

## Rasmussen et al. method

Rasmussen et al. (2014) (Supplement 17) considered the problem of whether an archaic genome was from a population directly ancestral to a present-day population. Like the TT method, two chromosomes are sampled from each population. The two populations *A* and *B* were assumed to have separated at some time in the past. To eliminate mutation as a force, they restricted their analysis to sites that were ascertained to be polymorphic in an outgroup, as is assumed in the TTo method of Sjödin et al. (2020). In fact, the two methods are equivalent but Rasmussen et al. (2014) restricted themselves to the specific question of direct ancestry.

We call the two alleles by S and s. Without distinguishing ancestral and derived states, there are five configurations of the data at each site: (1) SS/SS or ss/ss, (2) SS/Ss or ss/Ss, (3) SS/ss or ss/SS, (4) Ss/SS or Ss/ss, and (5) Ss/Ss, where the first genotype is from population *A* and the second is from *B*. Rasmussen et al. (2014) showed that, in the absence of mutations, the probabilities of the five configurations depend on five parameters, *c*_1_, the probability that the two lineages from *A* coalesce after the populations diverge, *c*_2_, the probability that the two lineages from *B* coalesce after the populations diverge, and *k*_0_, *k*_1_, and *k*_2_, the elements of the normalized folded site-frequency spectrum in a sample of size 4 immediately before the populations diverged: *k*_0_ is the probability of SSSS or ssss, *k*_1_ is the probability of SSSs or Ssss, and *k*_2_ is the probability of SSss, where the ordering of S and s does not matter.

The data consist of the numbers of sites *n*_i_ with each configuration. Rasmussen et al. (2014) assumed that the data had a multinomial distribution with probabilities *p*_*i*_. They used standard numerical methods to estimate the five parameters from the data. As with the *F*(*A*|*B*) and TT methods, this is a composite likelihood method because it assumes independence of sites that may be correlated because of linkage disequilibrium.

Rasmussen et al. (2014) applied their method to an archaic sample from Montana, which in this notation is population *B*, and several present-day Native American individuals, each of which in turn was population *A*. Rasmussen et al. restricted their analysis to sites that are polymorphic in a panel of African individuals. Rasmussen et al. used a likelihood ratio test of the hypothesis that *c*_2_ = 0. If *c*_2_ = 0, the branch to *B* from the population ancestral to *A* and *B* was so short that no coalescence events occurred, which implies that B is directly ancestral to A or nearly so. In doing this analysis, Rasmussen et al. (2014) needed no assumptions about the history of population sizes either before or after *T*. They did not estimate divergence times, only coalescence probabilities.

## *G*(*A*|*B*) method

In this paper, we simplify the Rasmussen et al. (2014) method and assume that only one chromosome is sampled from population *A*, as in the *F*(*A*|*B*) method. To emphasize the similarity to the *F*(*A*|*B*) method, we call our method the *G*(*A*|*B*) method. There is no need to assume that *A* is a present-day population or even that it was from a more recent time than *B*. The goal is to estimate the coalescence probability in *B* before *A* and *B* had a common ancestor. From that coalescence probability and assumptions about population size changes in *B*, we can estimate *T*, the time since *B* separated from the common ancestor.

With only one chromosome sampled from *A*, there are three configurations of the data: (1) S/SS or s/ss, (2) S/Ss or s/Ss, and (3) S/ss or s/SS, where the allele carried by the chromosome from A is before the slash. There are only three parameters of the model, *c*, the probability of coalescence in *B*, and *k*_0_ and *k*_1_, the elements of the normalized folded site-frequency spectrum in a sample of size 3 at *T*: *k*_0_ is the probability of SSS or sss and *k*_1_ is the probability of SSs or Sss. There are only two free parameters because *k*_0_ + *k*_1_ = 1. By analogy with the derivation in Rasmussen et al. (2014):

$$p_1 = k_0 + \frac{{ck_1}}{3}$$

6 $$p_2 = \frac{{2\left( {1 - c} \right)k_1}}{3}$$

$$p_3 = \frac{{2\left( {1 + c} \right)k_1}}{3}$$

where the *p*_*i*_ are the configuration probabilities. Given the data, *n*_*i*_ for *i* = 1, 2, 3, the three parameters can be estimated by assuming a multivariate normal distribution of the data.

The estimated value of *c* does not require any assumptions about population size but also provides no information about the divergence time. From $\hat c$, the estimate of *T* ($\hat T$) is obtained solving the equation

7 $$\hat c = 1 - {\rm{exp}}\left[ { - {\int}_0^{\hat T} {\frac{{dt}}{{2N\left( t \right)}}} } \right]$$

for $\hat T$ once an assumption is made about *N*(*t*). If *N* is constant $\hat T = - 2N{\rm{ln}}\left( {1 - \hat c} \right)$. In our application of this method, we used inferences about *N*(*t*) obtained from PSMC (Li and Durbin 2011) but other methods including historical data could be used instead. Differences among inferred historical population sizes will result in differences in estimated divergences times.

## Comparison of *G*(*A*|*B*) with other methods

We can understand the relationship to *F*(*A*|*B*) by assuming the sample sizes are large enough that the numbers of each configuration take their expected values. In that case, the parameter estimates for the *G*(*A*|*B*) method are

8 $$\hat c = \frac{{2n_3 - n_2}}{{2n_3 + n_2}}$$

9 $$\hat k_1 = \frac{3}{4}\frac{{2n_3 + n_2}}{n}$$

where *n* = *n*_1_ + *n*_2_ + *n*_3_.

The *F*(*A*|*B*) method is similar. To apply it, ancestral and derived alleles must be distinguished. Let S be the derived allele. There are six configurations of the data (1) S/SS, (2) S/Ss, (3) S/ss, (4) s/SS, (5) s/Ss, and (6) s/ss. Let *v*_*i*_ be the observed numbers of sites in each configuration. By definition,

10 $$F\left( {A|B} \right) = \frac{{v_2}}{{v_2 + v_5}}$$

When ancestral and derived alleles are not distinguished, *n*_1_ = *v*_1_ + *v*_6_, *n*_2_ = *v*_2_ + *v*_5_, and *n*_3_ = *v*_3_ + *v*_4_. Hence, from (8),

11 $$\hat c = \frac{{2\left( {v_3 + v_4} \right) - v_2 - v_5}}{{2\left( {v_3 + v_4} \right) + v_2 + v_5}}$$

There are several differences between the two methods. First, the two methods use different subsets of sites. *F*(*A*|*B*) uses all sites that are heterozygous in *B* while our method uses all sites that are polymorphic in an outgroup. Second, the *F*(*A*|*B*) method estimates *T* directly from simulations, while our method first estimates *c* and from that value estimates *T*. Given the assumptions about demography, *c* is an analytic function of *T* and the estimate of *T* is found analytically or numerically. No simulations are needed. Third, the estimate of *T* from our method does not depend on the history of population size in the ancestral population. That history determines *k*_1_ that is estimated from the data.

The TT method does not require assumptions about the sizes of the daughter populations but it does rely on the assumption that the ancestral population was of constant size and had reached an equilibrium under mutation and genetic drift.

The TTo method of Sjödin et al. (2020) is equivalent to our method except that it assumes that two chromosomes are sampled from each population. Their expressions for the coalescence probabilities are, in terms of the notation used here

$$\hat c_1 = 1 - 2\frac{{m_{1,0} + m_{1,2} + m_{1,1}}}{{2\left( {m_{1,0} + 2m_{2,0} + m_{2,1}} \right) + m_{1,1}}}$$

$$\hat c_2 = 1 - 2\frac{{m_{0,1} + m_{2,1} + m_{1,1}}}{{2\left( {m_{0,1} + 2m_{0,2} + m_{1,2}} \right) + m_{1,1}}}$$

where the *m*_*i,j*_ are the number of sites with *i*-derived alleles in population 1 and *j*-derived alleles in population 2.

## Test for treeness in three or more populations

When samples from three or more populations are available, estimates of coalescence probabilities can be obtained from all pairs. If the history of the populations is correctly represented by a bifurcating population tree in which there is no immigration either among the populations sampled or from an external population, then the coalescence probabilities are constrained because the probabilities on different branches of the population tree must be additive. As a consequence, it is possible to use our method to test whether the population history is treelike even when the history of population sizes is unknown. Our test differs from the test of treeness presented by Sjödin et al. (2020). Their test is of the hypothesis that the outgroup and two populations are correctly modeled by a bifurcating tree with no admixture. Our test is a test of the hypothesis that three populations form a bifurcating tree given that the sites are ascertained to be polymorphic in a population that is an outgroup to all three. Our test of treeness does not require assumptions about population sizes because only the coalescence probabilities are used.

**Fig. 2:**
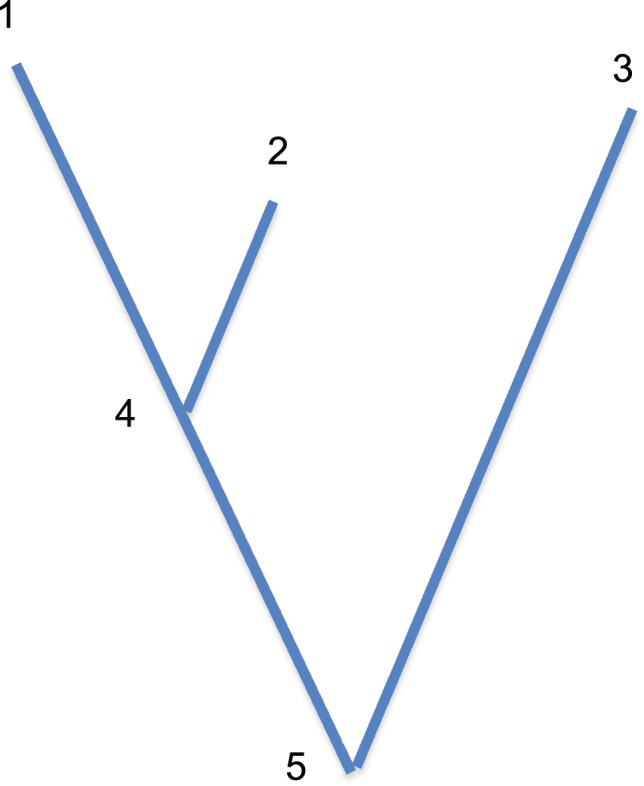
Illustration of a tree of three populations, 1, 2 and 3, used in the test of treeness described in the text. Population 4 is ancestral to 1 and 2, and population 5 is ancestral to all three populations.

**Fig. 3:**
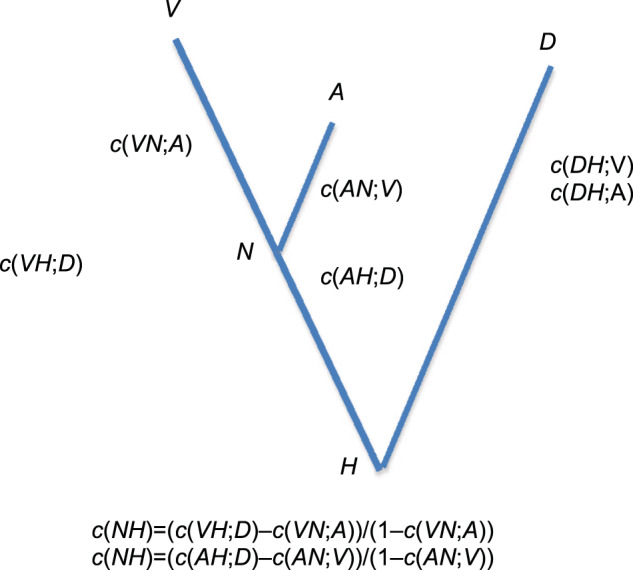
Application of the method described in the paper for estimating the coalescent probability, *c*, on each branch of a population tree of three archaic genomes. The six coalescence probabilities that can be estimated are shown.

We illustrate this idea with three populations, shown in Fig. 2. The samples are from populations 1, 2, and 3, which are not necessarily contemporaneous. The ancestral populations are 4 and 5. We distinguish coalescence probabilities on each branch by the identities of the initial and final populations, *c*(14), *c*(24), etc. We estimate each of these probabilities using two populations, one from which two chromosomes are sampled (population *B*) and the other from which a single chromosome is sampled (population *A*). We indicate the population used as population *A* in each estimate. For example $\hat c\left( {14;2} \right)$ is the estimate of *c*(14) using a single chromosome from population 2. One test of treeness comes from the two ways of estimating *c*(35), namely $\hat c\left( {35;1} \right)$ and $\hat c\left( {35;2} \right)$. If there is no admixture, these two estimates should be the same (Fig. 3). We define the test statistic, Δ_1_ to be the difference:

12 $$\Delta _1 = \hat c\left( {35;1} \right) - \hat c\left( {35;2} \right)$$

A second test of treeness comes from the fact that the coalescence probability on the internal branch, *c*(45), can be estimated two ways. A coalescence on branch 45 can occur only if there is no coalescence on one of the terminal branches, 14 or 24, and a coalescence on branch 15 or 25. Therefore

13 $$c\left( {45} \right) = \frac{{c\left( {15} \right) - c\left( {14} \right)}}{{1 - c\left( {14} \right)}} = \frac{{c\left( {25} \right) - c\left( {24} \right)}}{{1 - c\left( {24} \right)}}$$

If the population history is treelike, the second test statistic

14 $$\Delta _2 = \frac{{\hat c\left( {15;3} \right) - \hat c\left( {14;2} \right)}}{{1 - \hat c\left( {14;2} \right)}} - \frac{{\hat c\left( {25;3} \right) - \hat c\left( {24;1} \right)}}{{1 - \hat c\left( {24;1} \right)}}$$

will be 0.

A rough test of whether Δ_1_ and Δ_2_ differ significantly from 0 is obtained by approximating their variances using the variances in the values of $\hat c$. For example, the variance in Δ_1_ is the sum of the variances of $\hat c\left( {35;1} \right)$ and $\hat c\left( {35;2} \right)$, provided errors in those two quantities are assumed to be independently distributed. A slightly more elaborate expression is needed to compute the variance of Δ_2_ because of the denominators. From these variances, the hypothesis that Δ_1_ and Δ_2_ differ from 0 is rejected if the estimates values are more than two standard deviations from 0.

## Application to Neanderthals and Denisovans

We illustrate the application of our methods to three high-coverage archaic genomes, the Altai Neanderthal from the Denisova Cave in central Siberia (Prüfer et al. 2014), the Vindija Neanderthal from the Vindija Cave in Croatia (Prüfer et al. 2017), and the Denisova genome (Meyer et al. 2012). All three genomes were sequenced to sufficient depth that heterozygous sites can be called with confidence. Hence, the effects of degradation of aDNA do not affect the results.

In applications to lower coverage sequences, statistical uncertainly about homozygous and heterozygous sites would have to be taken into account by using genotype likelihoods. However, because estimates of coalescence probabilities depend on the difference between a relatively small number of heterozygous and homozygous sites (cf. Equation 8 and values of *n*_2_ and *n*_3_ in Table 1), substantial uncertainty in the numbers of heterozygous and homozygous sites in low coverage sequences would probably result in unacceptably large errors in estimated coalescence probabilities. As we will see in the present example, there is considerable uncertainly in the estimates of coalescence probabilities even when there is no uncertainty in the values of *n*_2_ and *n*_3_.

**Table 1:** Counts of SNPs in each of the three configurations along with estimates of *k*_1_ and *c*.

*B*	*A*	*n*_1_	*n*_2_	*n*_3_	*k*_0_	*c*
Altai	Vindija	7,864,107	75,478	57,311	0.0178 ± 0.001	0.206 ± 0.040
Vindija	Altai	7,874,698	54,704	67,494	0.0178 ± 0.001	0.423 ± 0.043
Vindija	Denisova	8,980,266	52,108	553,786	0.091 ± 0.002	0.910 ± 0.014
Denisova	Vindija	8,973,604	64,912	547,644	0.091 ± 0.003	0.888 ± 0.002
Altai	Denisova	7,923,816	78,910	465,399	0.089 ± 0.007	0.846 ± 0.056
Denisova	Altai	7,939,928	46,745	481,452	0.089 ± 0.002	0.907 ± 0.017

Both chromosomes are sampled from population *B* and one chromosome chosen at random is sampled from population *A*. Sites were ascertained to be polymorphic in 40 African individuals in the Simons Genome Diversity Panel (Mallick et al. 2016). *n*_1_, *n*_2_, and *n*_3_ are the numbers of sites in each of the three configurations defined in the text. *k*_0_ and *c* are obtained by assuming the *n*_*i*_ have a trinomial distribution with probabilities given by Eq. (4) in the text and maximizing the likelihood. The confidence intervals we obtained from a block-jackknife analysis using a window size of 10 mb.

We restricted our analysis to SNPs ascertained to be polymorphic in a panel of 40 African genomes in the Simons Genome Diversity Panel (Mallick et al. 2016). We used an additional filtering step for the Altai genome. Prüfer et al. (2014) showed that the Altai Neanderthal was inbred with an estimated inbreeding coefficient of 1/8. For the comparisons involving this individual, only sites not in runs of homozygosity longer than 2 mb were analyzed.

With three populations, there are six possible comparisons using each population in turn as population *A* and *B*. Table 1 shows the number of sites in each of the three configurations for all combinations. In the table, one of two alleles chosen at random from population *A* and two from population *B* were counted. The estimated value of *c* is the probability of coalescence in *B* after it diverged from the ancestor of *A* and *B*. The confidence intervals for *c* and *k*_1_ were obtained from block-jackknife resampling with a window size of 10 mb. The block-jackknife method is discussed by Green et al. (2010).

The two tests statistics defined in the previous section can be computed. In this context, population 1 is the Vindija Neanderthal, denoted by *V*, population 2 is the Altai Neanderthal, denoted by *A*, and population 3 is the Denisova genome, denoted by *D*. *N* denotes the common ancestor of *A* and *V* (population 4) and *H* denotes the common ancestor of all three populations (population 5). Adapting the notation in the previous section

$$\Delta _1 = \hat c\left( {DH;V} \right) - \hat c\left( {DH;A} \right) = 0.888 - 0.907 = - 0.019$$

and

$$\Delta _2 = \frac{{\hat c\left( {VH;D} \right) - \hat c\left( {VN;A} \right)}}{{1 - \hat c\left( {VN;A} \right)}} - \frac{{\hat c\left( {AH;D} \right) - \hat c\left( {AN;V} \right)}}{{1 - \hat c\left( {AN;V} \right)}}$$

$$= \frac{{0.910 - 0.423}}{{1 - 0.423}} - \frac{{0.846 - 0.206}}{{1 - 0.206}} = 0.038$$

For both test statistics, the numbers are taken from the last column of Table 1. Given the confidence intervals on the values of $\hat c$, Δ_1_ and Δ_2_ are not significantly different from 0.

**Fig. 4:**
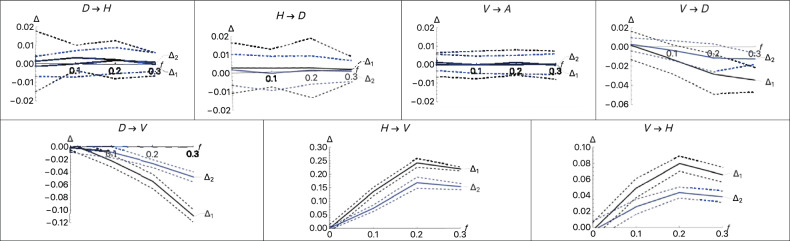
Results of simulations testing the sensitivity of the test for treeness to different types of introgression. In each part, the type of introgression is indicated. The simulation model assumed that the divergence of the two Neanderthal populations (node N) occurred 0.3 scaled time units ago, the divergence of Neanderthals and Denisovans (node H) occurred 0.6 scaled time units ago, and the divergence of humans with the ancestors of Neanderthals and Denisovans occurred 1 time unit ago. The solid lines indicate the averages of both test statistics (Δ_1_, black lines, and Δ_2_, blue lines) over ten replicates with 1,000,000 SNPs simulated. The dashed black and blue lines indicate the averages plus and minus two standard deviations across replicates. We assumed that a pulse of admixture occurred 0.1 scaled time units in the past. The parameter *f* is the fraction of the recipient population replaced by immigrants from the donor population.

To determine whether these test statistics are sensitive to deviations from a treelike population history, we conducted a simulation study tailored to this application of our test of treeness. We assumed that diploid sequences were sampled from *V*, *A*, and *D*, and five diploid sequences from an outgroup, denoted by *H*. We chose parameters to roughly agree with what is known about the history of Neanderthals, Denisovans, and modern humans, although we did not take the estimated ages of the fossils into account. We used the program scrm (Staab et al. 2015) to simulate SNP data under the assumption that sites are unlinked and neutral. In all results shown, we simulated ten replicates with 1,000,000 sites each. We set the time of the common ancestor of Altai and Vindija (node N) to be 0.3, the time of the common ancestor with the Denisovan (node H) to be 0.6 and the time of common ancestry with the outgroup to be 1, with all times measured in units of 2*N* generations. We allowed for admixture at time 0.1 at a rate *f* between various pairs of populations. Some results are shown in Fig. 4.

We can see that our test statistics are not sensitive to some kinds of admixture, supporting the idea that our estimates of coalescence probabilities are robust to small amounts of admixture. Admixture between Denisovan and the outgroup, *D*→*H* and *H*→*D*, has little effect even if *f* = 0.3. Admixture into Vindija either from Denisovan *D*→*V* or the outgroup *H*→*V* has a much larger effect, as does admixture to the outgroup *A*→*H*. Even in those cases, however, *f* has to be substantial for Δ_1_ and Δ_2_ to be strongly affected. It is already known that there was some admixture between Altai and Vindija and between a super-archaic group and Denisova (Prüfer et al. 2014) but both rates are low, on the order of 1%. Therefore, it is not surprising that the values of Δ_1_ and Δ_2_ are not large enough to indicate that the history of these three groups is not treelike. We have to conclude that our test of a treelike ancestry is not very powerful for detecting small amounts of admixture when only three populations are sampled. If our test does show deviations from a treelike history, however, the admixture levels required must be substantial or the wrong population tree is being assumed.

## Estimating divergence time from the coalescence probability

To convert the estimates of *c* to estimates of *T*, we need to solve Eq. (5) numerically after assuming something about the history of population sizes. We used the size estimates obtained by Prüfer et al. (2017) from applying PSMC (Li and Durbin 2011) to each genome. PSMC returns piecewise constant estimates, with size *N*_*i*_ in time interval (*t*_*i*_,*t*_*i*+1_) with *t*_0_ = 0. We used the time intervals and sizes reported in Figure S7.5 in Supplement 7 of Prüfer et al. (2017). We note that PSMC estimates an effective population size that includes the effects of admixture if there was any.

For piecewise constant population sizes, Eq. (5) reduces to

15 $$Pr\left( {c|T} \right) = 1 - e^{\left( {T - t_j} \right)/\left( {2N_j} \right)}\mathop {\prod}\limits_0^{j - 1} {e^{\left( {t_{i + 1} - t_i} \right)/\left( {2N_i} \right)}}$$

where *j* is chosen so that *t*_*j*_ < *T*≤*t*_*j*+1_. Solving Eq. (15) yields an estimate of *T*/(2*N*_0_), where *N*_0_ is different for different populations. For the Vindija and Altai branches, we obtained $\frac{{T_{VN}}}{{T_{AN}}} = 3.041$. This ratio is smaller than the ratio of 4 estimated by Prüfer et al. (2017). It is difficult to determine the cause of this difference.

The estimates of coalescence probabilities shown in Table 1 do not depend on assumptions of population history but the inferred divergence times do. That is a weakness of our method that is shared with the *F*(*A*/*B*) and TT methods. Different methods of inferring the history of population sizes will produce different estimates of *N*(*t*) in population *B*, which could then be used to assess the effects of different methods of inference.

## Discussion and conclusions

We present a simple method to estimate coalescence probabilities within population lineages and the divergence time of populations when single genomes are sampled from each population. Our method is a minor modification of a method introduced by Rasmussen et al. (2014). We compare the theoretical basis of our method with that of other methods, the *F*(*A*/*B*) method (Green et al. 2010) and the TT and TTo methods (Schlebusch et al. 2017; Sjödin et al. 2020). The three methods are similar in using SNP data from diploid genomes sampled from each population. They all analyze polymorphic SNPs as if they are unlinked. And they all assume a model in which two populations diverged from one another instantaneously at some time in the past and remained isolated until the genomic samples were taken. None of the methods assumes that the samples are taken at the same time and hence are all applicable to ancient DNA if it is of sufficient quality that heterozygous sites can be called accurately. To obtain estimates of divergence times they all require estimates of the per-site mutation rate.

The three methods differ slightly in the assumptions they make. The *F*(*A*/*B*) and TT methods assume that ancestral and derived alleles can be distinguished. Our method does not. The *F*(*A*/*B*) method and implicitly the TT method both require assumptions about the size of the ancestral population and the TT method assumes that the ancestral population was of constant size. The *F*(*A*/*B*) method assumes a history of population sizes inferred from PSMC (Li and Durbin 2011). The *G*(*A*|*B*) makes no assumption about the size of the ancestral population. The demography of the ancestral population is captured in the parameter *k*_1_ that characterizes the folded site-frequency spectrum at the time of population separation.

The three methods differ in which sites are analyzed. The *F*(*A*/*B*) method uses all sites that are heterozygous in one population (population *B*). The *G*(*A*|*B*) and TTo methods analyze all sites that are polymorphic in an outgroup. The TT method analyzes all sites polymorphic in the two genomes.

The three methods differ in how they estimate divergence times. Both the *F*(*A*/*B*) and TT methods estimate the divergence times scaled by the mutation rate. The *G*(*A*|*B*) method and implicitly the TTo method first estimate the coalescence probability in each population and then estimate the divergence time from assumptions about the history of population size after the populations diverged. In practice, the history of population sizes is inferred from PSMC or similar programs that assume a mutation rate. Therefore, all methods depend on an assumed mutation rate. None of the methods take account of variation in mutation rate across sites.

One goal of our paper is to call attention to several methods for estimating population divergence times using SNP data from pairs of genomes and to examine the relationship among them. These methods have a similar theoretical basis. The differences between them are relatively minor. Most important to the accuracy of results obtained using any of them is the assumption of complete isolation of the populations after they diverged from a common ancestor and the accuracy of the mutation rate and demographic history assumed.

## References

[CR1] Green RE, Krause J, Briggs AW, Maricic T, Stenzel U, Kircher M (2010). A draft sequence of the Neandertal genome. Science.

[CR2] Li H, Durbin R (2011). Inference of human population history from individual whole-genome sequences. Nature.

[CR3] Mallick S, Li H, Lipson M, Mathieson I, Gymrek M, Racimo F (2016). The Simons Genome Diversity Project: 300 genomes from 142 diverse populations. Nature.

[CR4] Meyer M, Kircher M, Gansauge M-T, Li H, Racimo F, Mallick S (2012). A high-coverage genome sequence from an archaic Denisovan individual. Science.

[CR5] Prüfer K, de Filippo C, Grote S, Mafessoni F, Korlević P, Hajdinjak M (2017). A high-coverage Neandertal genome from Vindija Cave in Croatia. Science.

[CR6] Prüfer K, Racimo F, Patterson N, Jay F, Sankararaman S, Sawyer S (2014). The complete genome sequence of a Neanderthal from the Altai Mountains. Nature.

[CR7] Rasmussen M, Anzick SL, Waters MR, Skoglund P, DeGiorgio M, Stafford TW (2014). The genome of a Late Pleistocene human from a Clovis burial site in western Montana. Nature.

[CR8] Schlebusch CM, Malmström H, Günther T, Sjödin P, Coutinho A, Edlund H (2017). Southern African ancient genomes estimate modern human divergence to 350,000 to 260,000 years ago. Science.

[CR9] Schlebusch CM, Skoglund P, Sjödin P, Gattepaille LM, Hernandez D, Jay F (2012). Genomic variation in seven Khoe-San groups reveals adaptation and complex African history. Science.

[CR10] Sjödin P, McKenna J, Jakobsson M (2021) Estimating divergence times from DNA sequences. Genetics 217:iyab008 10.1093/genetics/iyab008 PMC8049563 33769498

[CR11] Skoglund P, Götherström A, Jakobsson M (2011). Estimation of population divergence times from non-overlapping genomic sequences: examples from dogs and wolves. Mol Biol Evolution.

[CR12] Staab PR, Zhu S, Metzler D, Lunter G (2015). scrm: efficiently simulating long sequences using the approximated coalescent with recombination. Bioinformatics.

[CR13] Wakeley J (2009) Coalescent theory. Roberts & Company, Greenwood Village, Colorado

[CR14] Zuckerkandl E, Pauling L (1962) Molecular disease, evolution, and genetic heterogeneity. In: Kasha M, Pullman B (eds) Horizons in biochemistry. Academic Press, New York, p. 189–225

[CR15] Zuckerkandl E, Pauling L (1965) Evolution divergence and convergence in proteins. In: Bryson V, Vogel HJ (eds) Evolving genes and proteins. Academic Press, New York, p. 97–166

